# Association between Perceived Organizational Support for Infection Prevention and Work Engagement during the COVID-19 Pandemic among Japanese Workers: A Prospective Cohort Study

**DOI:** 10.3390/ijerph192316142

**Published:** 2022-12-02

**Authors:** Kiminori Odagami, Tomohisa Nagata, Kosuke Mafune, Hajime Ando, Seiichiro Tateishi, Mami Kuwamura, Ryutaro Matsugaki, Yoshihisa Fujino, Koji Mori, on behalf of the CORoNaWork Project

**Affiliations:** 1 Department of Occupational Health Practice and Management, Institute of Industrial Ecological Sciences, University of Occupational and Environmental Health, Kitakyushu 807-8555, Japan; 2 Department of Mental Health, Institute of Industrial Ecological Sciences, University of Occupational and Environmental Health, Kitakyushu 807-8555, Japan; 3 Department of Work Systems and Health, Institute of Industrial Ecological Sciences, University of Occupational and Environmental Health, Kitakyushu 807-8555, Japan; 4 Disaster Occupational Health Center, Institute of Industrial Ecological Sciences, University of Occupational and Environmental Health, Kitakyushu 807-8555, Japan; 5 Department of Environmental Health, School of Medicine, University of Occupational and Environmental Health, Kitakyushu 807-8555, Japan; 6 Department of Public Health, School of Medicine, University of Occupational and Environmental Health, Kitakyushu 807-8555, Japan; 7 Department of Environmental Epidemiology, Institute of Industrial Ecological Sciences, University of Occupational and Environmental Health, Kitakyushu 807-8555, Japan

**Keywords:** COVID-19, infection prevention, Japan, perceived organizational support, work engagement

## Abstract

Although the correlation between perceived organizational support (POS) and work engagement has been investigated in several studies, the relationship between health-focused POS and work engagement has not been clarified. We prospectively evaluated the influence of workers’ POS for infection prevention (POS-IP) on employees’ work engagement. This prospective cohort study was conducted from December 2020 (baseline) to December 2021 (1-year follow-up) using a self-administered internet questionnaire. At follow-up, there were 18,560 respondents, and after excluding 6677 respondents who had changed jobs or retired since baseline or who were self-employed; thus, 11,883 participants were included in the analysis. We asked participants a single question on POS-IP and the three-item Utrecht Work Engagement Scale (UWES-3), and then analyzed the relationship between POS-IP at baseline and UWES-3 at follow-up using multilevel regression analysis. Work engagement at follow-up was significantly higher in the groups with “low”, “high”, and “very high” POS-IP at baseline as compared with the “very low” group (all, *p* < 0.001). A dose-response relationship was also observed between the POS-IP categories at baseline and work engagement at follow-up (*p* for trend < 0.001). During the COVID-19 pandemic, POS-IP can increase work engagement after 1 year.

## 1. Introduction

Perceived organizational support (POS) is defined by global beliefs concerning the extent to which an organization values employees’ contributions and cares about their well-being [1]; a concept that emphasizes the employee’s perspective and how companies’ or organizations’ efforts and responses are valued by the people who work for them. Work engagement refers to “a positive fulfilling, affective, motivational state of work-related well-being, described by vigor, dedication, and absorption” [2]. Work engagement can be easily assessed using the well-known Utrecht Work Engagement Scale (UWES), developed by Schaufeli et al. The UWES has been standardized in various countries and has shown good results in both reliability and validity [3].

The correlation between POS and work engagement has been investigated in several studies, and POS is considered one of the most important factors promoting work engagement [3,4,5]. For example, Caesens et al. determined that POS is positively correlated with employee work engagement. Those authors also found that POS is positively correlated with the three dimensions of employee work engagement: vigor, dedication, and absorption [5]. If employees feel a high level of POS, their needs for autonomy and competence will be satisfied, which may lead to increased affective commitment, psychological capital, and psychological safety, which may increase work engagement [3,6,7,8].

Although POS is used to evaluate employees’ perceptions of organizational concern for well-being in general, even support for specific issues, such as POS for health and POS for health promotion, have been found to be related to employee presenteeism, self-rated health, and COVID-19 vaccine coverage [9,10,11,12]. Furthermore, Mihalache et al. found that POS, in relation to the COVID-19 pandemic, improves organizational commitment and job-related well-being [13]. However, to our knowledge, no studies have clarified the relationship between health-focused POS (such as POS for health or POS regarding the COVID-19 pandemic) and work engagement.

We hypothesized that among health-focused POS, POS for infection prevention (POS-IP) would have a positive impact on work engagement. In the context of the COVID-19 pandemic, infection prevention in the workplace protects the health and lives of employees and is considered one of the highest priority organizational supports for employees. In this study, we prospectively assessed the influence of employees’ POS-IP on employees’ work engagement by analyzing data from the Collaborative Online Research on the Novel-coronavirus and Work (CORoNaWork) Project.

## 2. Materials and Methods

### 2.1. Participants

This was a prospective cohort study conducted from December 2020 (baseline survey) to December 2021 (follow-up survey). Data for both the baseline and follow-up surveys were collected using a self-administered online questionnaire delivered by an internet survey company, Cross Marketing Inc. (Tokyo, Japan). The protocol for the CORoNaWork study has been previously reported in detail [14]. The participants included workers aged 20–65 years, at baseline, with sampling according to sex, occupation, and area of residence. In all, 33,087 participants were recruited; after excluding 6051 who provided invalid responses, we included 27,036 participants at baseline. We used the following criteria to determine invalid responses: extremely short response time (≤6 min), extremely low body weight (<30 kg), extremely short height (<140 cm), inconsistent answers to similar questions throughout the survey (e.g., inconsistency regarding questions about marital status and living situation), and wrong answers to a question meant to identify fraudulent responses (choose the third largest number from among five numbers).

**Figure 1:**
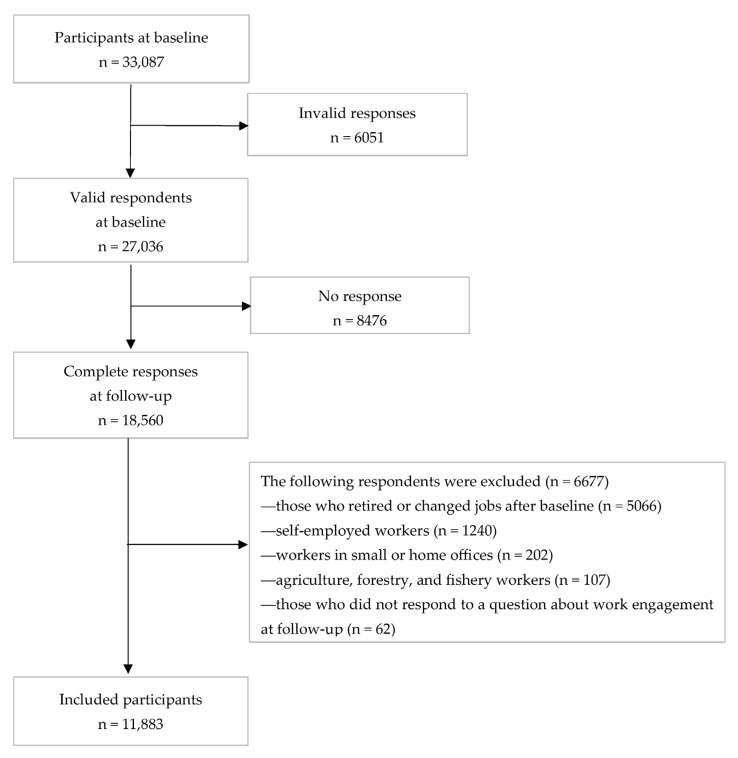
Flow chart for the study.

Participants received a follow-up survey in December 2021, 1 year after baseline. In all, 18,560 participants (tracking rate: 68.7%) responded to the follow-up survey. The exclusion criteria for the present study were as follows: self-employed workers; workers in small or home offices; agriculture, forestry, and fishery workers; and participants who retired or changed jobs after the baseline survey. Participants who failed to respond to a question about POS-IP at follow-up were also excluded. Finally, 11,883 participants were included in the analysis (Figure 1).

The present study was approved by the Ethics Committee of the University of Occupational and Environmental Health, Japan (reference No. R2-079 and R3-006). Informed consent was obtained through the CORoNaWork study survey website at the time the data were collected.

### 2.2. Assessment of Work Engagement 

Work engagement was assessed with the three-item short version of the Utrecht Work Engagement Scale (UWES-3) [15]. The UWES-3 has been validated in five countries, including Japan [15]; the scale measures vigor with one item (“At my work, I feel bursting with energy”), dedication with one item (“I am enthusiastic about my job”), and absorption with one item (“I am immersed in my work”). All items were rated using a seven-point Likert scale ranging from 1 = strongly disagree to 7 = strongly agree. In the baseline and follow-up sample, Cronbach’s alpha of UWES-3 (total score) was 0.92 and 0.89, respectively.

### 2.3. Assessment of POS for Infection Prevention (POS-IP) at Baseline

POS-IP was evaluated with the following item: “My company has adequate infection control measures for employees.” Participants answered using a four-point scale: strongly agree, agree, disagree, and strongly disagree. Responses were categorized as indicating very high, high, low, and very low POS-IP. The analysis was also performed with POS-IP as an ordinal variable. We defined a score of 4 for very high POS-IP, 3 for high POS-IP, 2 for low POS-IP, and 1 for very low POS-IP.

### 2.4. Assessment of Covariates

Covariates included demographic and socioeconomic factors, occupation industry, number of employees in the workplace, and infection control measures in the workplace. Age was expressed as a continuous variable. Yearly household income was classified into four categories: <2.50 million Japanese yen (JPY); 2.50–3.74 million JPY; 3.75–4.99 million JPY; and ≥5 million JPY. Education was classified into five categories: junior high school, high school, junior college or technical school, university, and graduate school. Marital status was classified into three categories: married; divorced or widowed; and unmarried. In this survey, participants chose 1 of 11 options for their occupation: general employee; manager; executive manager; public employee, faculty member or non-profit organization employee; temporary/contract employee; self-employed; small office/home office; agriculture, forestry, or fishing; professional occupation (e.g., lawyer, tax accountant, medical-related); and other occupations. As mentioned above, three of these categories were excluded in this study, such that occupations were finally classified into seven categories. Participants also chose one from among 22 options to describe the industry in which they worked: energy, materials, industrial machinery; food; beverages/tobacco products; pharmaceuticals/medical supplies; cosmetics/toiletries/sanitary products; fashion and accessories; precision machinery and office supplies; home appliances/audio visual equipment; automobiles and transportation equipment; household goods; hobbies/sporting goods; real estate and housing equipment; information and communication; distribution and retail; finance/insurance; transportation and leisure; restaurant and other services; public officers and organizations; education, medical services, religion; mass media; market research; and other. The number of employees in the workplace was classified into four categories: 1–9, 10–99, 100–999, and ≥1000 employees. Participants were asked, at baseline to choose whether each of the following eight workplace infection control measures, with the exception of the item related to telecommuting, was implemented in their workplace, with reference to a previous study [12]: (1) prohibition/restriction of business trips; (2) prohibition/restriction of visitors; (3) prohibition of holding, or limiting the number of people participating in, social gatherings and banquets; (4) restrictions on face-to-face meetings; (5) requirement to always wear a mask during working hours; (6) installation of partitions and change of workplace layout; (7) recommendation for daily temperature checks; and (8) request not to come to work when sick. The implementation status was expressed as the total number of measures implemented among the eight infection control measures.

The cumulative infection rate of COVID-19, determined in the participants’ prefecture of residence one month before the survey, was used as a community-level variable. This information was collected from public agency websites.

### 2.5. Statistical Analysis

To estimate whether POS-IP at baseline was associated with work engagement at follow-up among participants, we used a multilevel regression analysis nested in the prefecture of residence to account for area variability. The multivariate model was adjusted for sex and age (Model 1), and then additionally adjusted for equivalent income (categorical); educational background (categorical); marital status, occupation, working industry, number of employees in the workplace (categorical); and work engagement score at baseline (continuous) (Model 2). Finally, the model was additionally adjusted for the number of infection control measures in the workplace at baseline (continuous) (Model 3). All analyses used the infection rate of COVID-19 by prefecture as a community-level variable. Additionally, the *p*-values of multilevel regression analysis were calculated by considering each category scale of POS-IP as a continuous variable (*p* for trend). A *p*-value < 0.05 was considered statistically significant. All analyses were conducted using Stata version 16 (StataCorp LLC, College Station, TX, USA).

## 3. Results

Table 1 shows participants’ characteristics by POS-IP category at baseline. The group with higher POS-IP tended to be married, have higher income, and have a higher number of workplace infection control measures at baseline. The mean (standard deviation) UWES-3 score at follow-up was lowest at 1.5 (1.5) for very low POS-IP and the highest at 2.9 (1.6) for very high POS-IP. 

Table 2 shows the association between POS-IP at baseline and work engagement score at follow-up. In Model 1 (adjusted for sex and age) and Model 2 (Model 1 and additionally adjusted for demographics, including socioeconomic factors, occupation, industry, company size, and number of infection control measures in the workplace), scores of the “low”, “high”, and “very high” groups were significantly higher than those of the “very low” group (all *p* < 0.001). This significant association between POS-IP at baseline and work engagement at follow-up remained after adjusting for the work engagement score at baseline (Model 3). We also observed a dose-response relationship between the categories of POS-IP at baseline and work engagement score at follow-up (*p* for trend <0.001).

## 4. Discussion

In this study, we analyzed how POS-IP with respect to COVID-19 affected work engagement at 1-year follow-up. The results showed that the higher the POS-IP, the higher the work engagement after 1 year. These results support our hypothesis that high POS-IP and POS specific to infection control in the workplace have a positive effect on work engagement.

The present findings reveal a positive relationship between work engagement and employees’ perception that their workplace has adequate infection control measures (POS-IP) and a health-focused POS. Our findings are also consistent with those of previous research showing that POS is a key factor that promotes work engagement [4,8,16,17]. It has been shown that if employees feel a high level of POS, their needs for autonomy and competence will be satisfied, which leads to increased affective commitment, psychological capital, and psychological safety, which may increase work engagement [3,6,7,8]. This relationship between POS and work engagement supports the job demands–resources (JD-R) model, which assumes that job resources produced by the work environment, events, and actions affect work engagement [18]; POS is considered a job resource, given its definition [1]. In other words, employees’ perception that the workplace has adequate infection control measures in place during the pandemic (POS-IP) may play a similar role in POS, as a job resource, leading to improved work engagement.

To increase POS-IP and work engagement, workplace infection control measures need to be improved. Our previous studies have shown that health support for employees through proactive workplace infection control improves POS and work engagement [19,20]. In the present study, however, a significant direct relationship between POS-IP and work engagement remained after adjusting for the actual infection control status of the workplace. This suggests the importance of employees’ perception that infection control measures are well-implemented in the workplace (i.e., they feel that they receive sufficient support from their employer), in addition to actually implementing infection control measures. Existing research indicates that the extent to which workplace experiences contribute to POS depends on employees’ perception of organizational initiatives as discretionary rather than being driven by constraints, such as government regulation or union contracts [21]; additionally, no matter how favorable the response, if it is driven by external constraints, it has little effect on POS [22]. In other words, if employees perceive that the purpose of infection control in the workplace is to meet external constraints, such as requests from the government or local authorities, the implementation of these measures in the workplace may not contribute to improving POS-IP, and as a result, work engagement may not improve. Organizations must pay greater attention to how they communicate messages and when measures are taken to ensure that employees perceive that the measures against infectious diseases are being adequately implemented in the workplace, not out of a sense of obligation but out of genuine concern for the well-being of employees.

Four limitations exist in this study. First, because this study was an internet-based survey, individuals without internet access or who were not registered as monitors were omitted from the target population. However, because sampling bias was reduced by randomly sampling by region, occupation, and prefecture according to the incidence of COVID-19, the generalizability of our study results should be adequate. Second, the timing of the survey may have influenced the responses among participants. The baseline survey for this study was conducted in December 2020, when the number of COVID-19 infections in Japan was rapidly increasing, and the follow-up survey was conducted in December 2021, when infections were under control. Therefore, trends in the number of infections at the time of the survey may have affected perceptions of infection prevention measures in the workplace and work engagement. Third, it is difficult to conduct an analysis that takes into account changes in POS-IP from baseline. Although it is likely that the infection situations surrounding the survey participants (including whether the survey participants themselves had experienced infection) and infection control measures in their workplace fluctuated during the year between the baseline and follow-up surveys, it is unclear how and at what point the POS-IP changed. Fourth, POS-IP was assessed using original questions, and the measurement validity of the original POS-IP concept is untested; further research is needed to rigorously validate the POS-IP indicators.

## 5. Conclusions

The results of the present study suggest that higher perceived workplace infection control during the COVID-19 pandemic is associated with higher work engagement after 1 year. In addition, a significant direct relationship between POS-IP and work engagement remained after adjusting for infection control status in the workplace at baseline. Therefore, implementing appropriate workplace infection control measures during an infectious disease outbreak and supporting employees’ perception that those measures are well-implemented may positively impact employee work engagement.

## Figures and Tables

**Table 1:** Characteristics of participants by POS-IP category at baseline.

		Very Low	Low	High	Very High
Number of participants		975	2137	6741	2030
Age (y), mean (SD)		47.2 (8.8)	47.0 (9.6)	47.7 (9.7)	47.7 (10.2)
Sex					
	Male	543 (55.7%)	1269 (59.4%)	3838 (56.9%)	1022 (50.3%)
Yearly household income (million JPY)					
	<2.50	258 (26.5%)	376 (17.6%)	1080 (16.0%)	300 (14.8%)
	2.50–3.74	288 (29.5%)	658 (30.8%)	1796 (26.6%)	470 (23.2%)
	3.75–4.99	212 (21.7%)	587 (27.5%)	1766 (26.2%)	493 (24.3%)
	≥5.00	217 (22.3%)	516 (24.1%)	2099 (31.1%)	767 (37.8%)
Educational background					
	Junior high school	20 (2.1%)	27 (1.3%)	67 (1.0%)	20 (1.0%)
	High school	324 (33.2%)	554 (25.9%)	1669 (24.8%)	450 (22.2%)
	Junior college/technical school	210 (21.5%)	449 (21.0%)	1432 (21.2%)	487 (24.0%)
	University	374 (38.4%)	1000 (46.8%)	3115 (46.2%)	951 (46.8%)
	Graduate school	47 (4.8%)	107 (5.0%)	458 (6.8%)	122 (6.0%)
Marital status					
	Married	474 (48.6%)	1205 (56.4%)	3935 (58.4%)	1217 (60.0%)
	Divorced or widowed	126 (12.9%)	187 (8.8%)	588 (8.7%)	211 (10.4%)
	Unmarried	375 (38.5%)	745 (34.9%)	2218 (32.9%)	602 (29.7%)
Occupation					
	General employee	606 (62.2%)	1212 (56.7%)	3330 (49.4%)	858 (42.3%)
	Manager	74 (7.6%)	243 (11.4%)	894 (13.3%)	258 (12.7%)
	Executive manager	16 (1.6%)	52 (2.4%)	300 (4.5%)	105 (5.2%)
	Public employee, faculty member, or non-profit organization	96 (9.8%)	277 (13.0%)	939 (13.9%)	245 (12.1%)
	Temporary/contract employee	84 (8.6%)	221 (10.3%)	704 (10.4%)	181 (8.9%)
	Professional occupation (lawyer, tax accountant, medical)	53 (5.4%)	95 (4.4%)	412 (6.1%)	295 (14.5%)
	Other occupation	46 (4.7%)	37 (1.7%)	162 (2.4%)	88 (4.3%)
Number of employees in the workplace					
	1–9	216 (22.2%)	306 (14.3%)	898 (13.3%)	306 (15.1%)
	10–100	329 (33.7%)	673 (31.5%)	1754 (26.0%)	454 (22.4%)
	100–999	231 (23.7%)	606 (28.4%)	1901 (28.2%)	613 (30.2%)
	≥1000	199 (20.4%)	552 (25.8%)	2188 (32.5%)	657 (32.4%)
Number of infection control measures in the workplace, mean (SD)		2.9 (2.7)	4.2 (2.6)	5.6 (2.4)	6.3 (2.1)
Work engagement (UWES-3; range: 0–6), mean (SD)		1.5 (1.5)	2.0 (1.4)	2.5 (1.4)	2.9 (1.6)

Abbreviation: POS-IP, Perceived Organizational Support for Infection Prevention; UWES-3, three-item Utrecht Work Engagement Scale; SD, standard deviation. Values are number (%) unless otherwise noted.

**Table 2:** Association between POS-IP at baseline and work engagement at follow-up.

	Model 1			Model 2			Model 3		
	Coef.	95% CI	*p*-Value	Coef.	95% CI	*p*-Value	Coef.	95% CI	*p*-Value
Perceived organizational support for infection prevention									
Very low	ref		<0.001 †	ref		<0.001 †	ref		<0.001 †
Low	0.51	0.41–0.62	<0.001	0.44	0.34–0.55	<0.001	0.16	0.08–0.24	<0.001
High	0.95	0.86–1.04	<0.001	0.80	0.70–0.89	<0.001	0.30	0.22–0.37	<0.001
Very high	1.40	1.29–1.50	<0.001	1.17	1.06–1.28	<0.001	0.46	0.37–0.54	<0.001

Model 1: adjusted for sex and age. Model 2: Model 1 and additionally adjusted for equivalent income (categorical), educational background (categorical), marital status, occupation, work industry, number of employees in the workplace, and number of infection control measures in the workplace (continuous). Model 3: Model 2 and additionally adjusted for work engagement score at baseline (continuous). Abbreviation: POS-IP, Perceived Organizational Support for Infection Prevention; Coef., coefficient; CI, confidence interval; ref, reference. † *p* for trend.

## Data Availability

The data presented in this study are available on request from the corresponding author. The data are not publicly available due to privacy.
